# In Situ Oil–Gas Separator Enabled Carrier-Free Photoacoustic Sensing of Acetylene

**DOI:** 10.3390/s26030946

**Published:** 2026-02-02

**Authors:** Weitao Dou, Xitong Sun, Yanping Gao, Shudong Wang, Kai Tao, Yunjia Li

**Affiliations:** 1 School of Electrical Engineering, Xian Jiaotong University, Xi’an 710049, China; dou_wt@stu.xjtu.edu.cn (W.D.); sunxitong@stu.xjtu.edu.cn (X.S.); gao.yan.ping.y@stu.xjtu.edu.cn (Y.G.); 2 Tianfu Jiangxi Laboratory, Chengdu 641419, China; 3 School of Mechanical Engineering, Northwestern Polytechnical University, Xi’an 710072, China; taokai@nwpu.edu.cn

**Keywords:** photoacoustic gas sensing, dissolved gas in oil, transformer diagnosis, lock-in amplifier

## Abstract

**Highlights:**

**What are the main findings?**

- A real-time photoacoustic spectroscopy system, integrated with a custom-designed multilayer oil–gas separation membrane, was developed for sensitive acetylene detection in transformer oil.
- The proposed system demonstrates a rapid response and high accuracy in continuous, in situ monitoring of dissolved gases, outperforming conventional off-line methods.

**What are the implications of the main findings?**

- The new approach enables timely transformer fault diagnosis, enhancing the reliability and safety of power equipment.
- This work lays a foundation for future development of compact and efficient online dissolved gas analysis (DGA) solutions for industrial applications.

**Abstract:**

In this work, a carrier-free photoacoustic spectroscopy system is developed for the detection of trace acetylene gas in insulating oil. The photoacoustic cell was integrated with an oil–gas separator, allowing dissolved gases in oil to be introduced into the cell through free diffusion. The oil–gas separator is a custom-fabricated AF2400-coated ceramic membrane, and its spin-coating process was carefully designed to enable rapid oil–gas separation and achieve high film flatness. Using a resonant photoacoustic cell and a low-noise lock-in amplifier, the sensitivity of the system was improved to 6.90 mV/ppm, with a repeatability error less than 1.65%. Calibration experiments demonstrated that continuous detection of dissolved gas in oil could be achieved, with a response time *T*_90_ of less than 72.5 min. Compared to traditional photoacoustic spectroscopy, the continuous measurement capability of this method is expected to enable earlier fault diagnosis, thus having greater potential in industrial fields.

## 1. Introduction

Oil-immersed transformers are critical components in power systems [1], and their long-term stable operation is essential for the power system [2]. Dissolved gas analysis (DGA) is widely regarded as the gold standard for monitoring transformer health [3]. According to the types and concentrations of dissolved gases in insulating oil, the faults of the transformer can be determined [4]. For instance, overheating of the oil results in the release of CH_4_ and C_2_H_4_ [5], partial discharge between oil and paper generates CH_4_, C_2_H_4_, CO, and CO_2_ [6], while arc discharge within the oil primarily produces H_2_ and C_2_H_2_ [7]. According to the IEEE guidelines for the interpretation of gases generated in mineral oil-immersed transformers, among these gases, the concentration of C_2_H_2_ is particularly significant for transformer fault diagnostics.

A variety of techniques have been developed for detecting dissolved gases in transformer oil, including gas chromatography [8], Raman spectroscopy [9], non-dispersive infrared technology [10], and photoacoustic spectroscopy (PAS) [11]. Among these methods, PAS systems determine gas concentrations by detecting sound waves generated when gas molecules absorb modulated infrared light [12], and thus offer advantages such as high sensitivity, a fast response, and infrequent calibration needs [13]. Therefore, PAS has been widely applied in online DGA systems [14]. However, in typical online DGA systems, PAS sensors are often used in combination with dynamic headspace degassing [15] or vacuum degassing methods to extract the dissolved gases from the transformer oil. In these configurations, an oil pump delivers the oil sample to a degassing unit, and a gas pump subsequently transports the extracted gases to the PAS sensor [16]. While effective, these methods are slow—producing only one measurement data per operational cycle—and demand large amounts of carrier gas. This increases operational complexity and cost, and limits their suitability for real-time monitoring applications.

Therefore, membrane-based oil–gas separation has emerged in recent years as a promising alternative to traditional degassing approaches for DGA [17]. Compared with conventional techniques, membrane separators offer continuous degassing without oil loss [18], reduced energy consumption, and cost-effectiveness [19]. Their feasibility for in situ measurements has been demonstrated in Raman spectroscopy [20], infrared spectroscopy [21], and PAS systems [22]. Despite these advances, most reported integrations still rely on commercially available organic membranes whose degassing time can be many hours. To enhance performance, numerous studies have explored material and process innovations, e.g., fluorinated ethylene–propylene (FEP) [23], polytetrafluoroethylene (PTFE) [17], and polydimethylsiloxane (PDMS) membranes [24]. Moreover, advances in high-temperature-stable crystal-based wireless modules provide a materials basis for real-time wireless DGA [25,26]. Yet, even with such efforts, the separation efficiency remains below that of vacuum degassing [27], which hinders their suitability for real-time, on-site monitoring applications [28].

In this work, a custom-made oil–gas separation membrane with multilayers is introduced. The separator is implemented with a resonant PAS gas-sensing system, thereby achieving in situ measurement of dissolved gas in insulating oil. The structure and dimensions of the photoacoustic cell were optimized through finite element simulation. A laser modulation circuit and a photoacoustic signal detection circuit, both based on a lock-in amplifier, were designed and integrated with the PAS cell. According to experimental results, continuous and highly sensitive acetylene detection was achieved by this system. The proposed system has the potential to greatly simplify the structure of transformer online DGA solutions, improve detection speed and accuracy, and provide a new path for real-time monitoring and intelligent maintenance of transformer status.

## 2. Design of the Proposed PAS System

### 2.1. Concept of the System

The working principle of the PAS gas-sensing system originates from the Beer–Lambert law and has been systematically described and validated in a wide range of photoacoustic gas-sensing studies [29]. When mid-infrared light is absorbed by gas molecules, a portion of the molecules in the ground state may transition to higher energy states. These excited molecules can return to their ground state via two pathways: radiative and non-radiative relaxation. The radiative path involves only the absorption and emission of photons, while the non-radiative path generates heat. If this process occurs within a confined space, i.e., the photoacoustic cell, it leads to the generation of acoustic pressure waves. When the infrared light is modulated at a specific frequency, an acoustic signal of the same frequency is produced. If this signal matches the cell’s eigenfrequency, the amplitude of the photoacoustic signal can be greatly enhanced.

**Figure 1:**
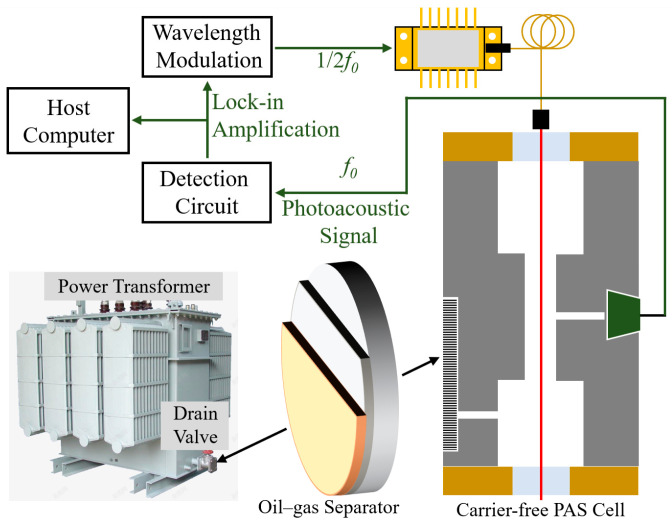
Concept of the PAS system.

Prior studies have integrated fiber-optic photoacoustic sensors with organic membrane separators, enabling the extraction and detection of dissolved gases [23]. To further reduce the response time of the system, this work designs a multilayer oil–gas separator that is directly coupled to the PAS cell. The concept of the carrier-free PAS gas-sensing system proposed in this paper is illustrated in Figure 1. The system consists of an oil–gas separator, a carrier-free PAS cell, a butterfly-packaged laser, and a modulation circuit. In practical applications, the oil–gas separator can be installed on the drain valve of a transformer. One side of the separator is in direct contact with transformer oil, and the other side interfaces with the PAS system’s gas chamber. In this way, the dissolved gases in the oil can directly diffuse into the PAS cell.

**Figure 2:**
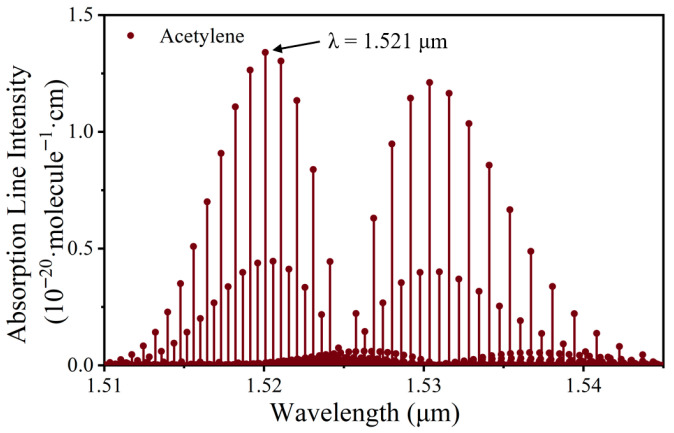
Absorption line intensity of acetylene.

The PAS cell adopts a typical H-shaped structure, through which the laser beam is directly transmitted. The laser used in this work has a center wavelength of 1.521 μm. According to the HITRAN database, acetylene gas exhibits a strong absorption line at 1.521 μm, with an absorption line intensity of 1.3 × 10^−20^ cm/molecule, as shown in Figure 2. In addition, this wavelength is close to the standard optical communication band, so laser sources and components such as collimators are more reliable and inexpensive.

When target gas is present in the photoacoustic cell, it interacts with the modulated laser light and creates an acoustic pressure signal. This pressure signal is amplified by the acoustic resonator, picked up by a microphone, and then processed by the detection circuit. The signal is further amplified and filtered by a lock-in amplifier, and finally sent to the computer for gas concentration analysis. This setup provides accurate and reliable gas detection without the need for a carrier gas.

### 2.2. Modeling and Design of the PAS Cell

When the modulated infrared beam enters the photoacoustic cell, the absorption of light by gas molecules results in local heating, which can be described by the power density of a heat source in the gas:

(1) $$v^{2}\nabla^{2}p(\vec{r},t)-\frac{\partial^{2}p(\vec{r},t)}{\partial t^{2}}=(\gamma -1)\frac{\partial H(\vec{r},t)}{\partial t}$$

where *v* is the speed of sound in the gas, *γ* is the heat capacity ratio of the gas, *H* is the volumetric thermal power density generated by absorbed radiation, and *p*(*r*,*t*) is the calculated sound pressure distribution within the photoacoustic cell.

By solving this non-homogeneous wave equation, the steady-state amplitude of the acoustic pressure at the microphone position can be expressed as

(2) $$p(\omega )=\frac{A_{0}}{\sqrt{{\left({\omega_{0}}^{2}-\omega^{2}\right)}^{2}+{\left(\omega_{0}\omega /Q_{0}\right)}^{2}}}$$

where *ω* is the modulation angular frequency of the incident light, *ω*_0_ is the first eigenfrequency of the photoacoustic cell, *Q*_0_ is the quality factor, and *A*_0_ is the amplitude given by

(3) $$A_{0}=\frac{(\gamma -1)\alpha IQ_{0}}{V}$$

where *V_c_* is the effective volume of the resonator, *α* is the absorption coefficient of the target gas, *I* is the incident optical power, and *V* is the total cell volume. The response of the acoustic sensor to this pressure signal is quantified by

(4) $$A_{s}(\omega )\propto I\cdot \alpha \cdot \frac{1}{V}\cdot S\cdot Q_{0}$$

where *S* is the sensitivity of the acoustic sensor.

Equation (4) describes how the amplitude of the frequency-dependent photoacoustic signal is analytically related to different geometrical parameters of the cell. Specifically, the amplitude increases with optical power, acoustic sensor sensitivity, quality factor, and the gas absorption coefficient, but decreases as the volume of the photoacoustic cell increases.

To further investigate the influence of the cell’s geometry and the engine frequency, a finite element model of the PAS sensor was developed using COMSOL Multiphysics^®^ 6.2. The thermal viscous acoustics and pressure acoustics modules were employed to develop the simulation model. The photoacoustic cell adopts a typical H-shaped geometry, comprising two buffer chambers and a linear acoustic resonator.

Its eigenfrequency is inversely proportional to the length of the resonator, while the generated sound pressure is also inversely proportional to the diameter. However, if the tube diameter is too small, scattered particles from the Gaussian beam may make contact with the sidewall, resulting in increased thermal background noise. The buffer chamber serves to reduce acoustic interference caused by freely diffusing gas flows. However, an over-large may result in an excessively bulky photoacoustic cell, ultimately affecting the response speed of the carrier-gas-free PAS system. In the design, the resonator has a diameter of 6 mm and a length of 2.4 cm, while each buffer chamber is 5 cm in diameter and 1.8 cm in length. A Gaussian beam is set to pass through the center of the photoacoustic cell. The gas absorption of the Gaussian beam and the resulted heat generation are simplified as a distributed heat source along the beam path.

**Figure 3:**
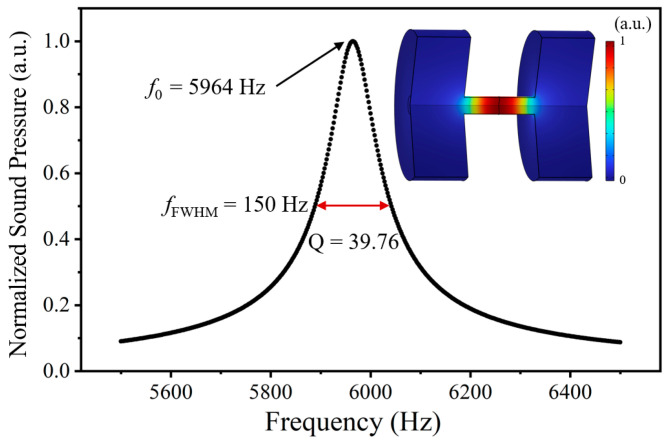
Normalized acoustic response of the photoacoustic cell.

Figure 3 presents the normalized acoustic response of the photoacoustic cell during a modulation frequency sweep of the Gaussian beam. The results reveal an eigenfrequency of 5964 Hz. The full width at half maxima (FWHM) is found to be 150 Hz, corresponding to a quality factor of 39.76. At resonance, the maximum sound pressure is observed at the center of the acoustic resonator. Therefore, to achieve optimal sensitivity, the microphone should be positioned at the center of the resonator during device assembly.

### 2.3. Design of the Oil–Gas Separator

The oil–gas separator provides the gas sample for the online DGA system by allowing dissolved gases in transformer oil to diffuse into the PAS cell. Consequently, the separator must satisfy two conflicting requirements: (i) achieving high extraction efficiency to ensure a fast system response, and (ii) withstanding elevated pressures because it is in direct contact with the oil.

**Figure 4:**
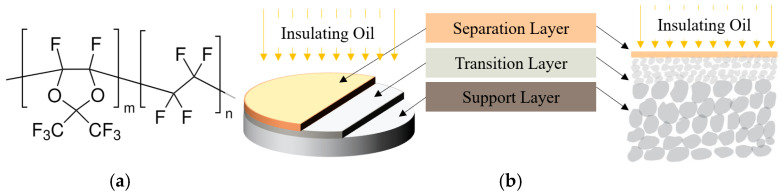
(**a**) Chemical structure of the amorphous fluoropolymer Teflon AF2400, (**b**) design of the oil–gas separation module.

To meet the first requirement for high permeability, Teflon AF2400 (Chemours, Wilmington, DE, USA) was selected as the functional separation material. As illustrated in Figure 4a, the polymer backbone contains rigid dioxole rings that prevent efficient chain packing. This structural rigidity, combined with weak inter-chain van der Waals forces, results in an exceptionally high fractional free volume, yielding superior gas permeability compared to semi-crystalline polymers [30]. Furthermore, the high bond energy of the carbon–fluorine (C-F) bonds imparts excellent thermal stability and chemical inertness, ensuring strong resistance to oil fouling [31].

Given that the intrinsic permeability of the material is fixed, reducing the thickness of the AF2400 layer is the primary means of shortening the oil–gas separation time. However, an ultra-thin polymer film alone lacks sufficient mechanical strength to withstand oil pressure. To overcome this limitation, a hierarchical α-Al_2_O_3_ ceramic substrate is employed.

The schematic cross-section shown in Figure 4b illustrates the resulting composite structure. Specifically, the membrane consists of an ultra-thin AF2400 selective layer supported by an asymmetric ceramic substrate. The substrate comprises a 25 μm thick transition layer with an average pore size of 100 nm, deposited on a 2 mm thick macroporous support layer with a pore size of 500 nm; both layers exhibit a porosity of 38%.

This gradient architecture serves a dual function. On the one hand, the transition layer provides a smooth and dense surface that prevents penetration of the polymer solution into the substrate, thereby enabling the formation of a defect-free selective layer. On the other hand, the macroporous support layer offers high mechanical robustness to withstand elevated hydraulic pressures while introducing minimal mass transfer resistance, effectively shortening the gas diffusion path through the membrane.

This multilayer architecture integrates the high gas permeability of the fluoropolymer with the mechanical robustness of the inorganic ceramic substrate. By simultaneously ensuring a high coating integrity and minimizing mass transport resistance, the design enables a continuous and stable supply of analyte gas for PAS measurements. As a result, both the detection sensitivity and response speed are markedly improved, fully satisfying the stringent reliability requirements for online power transformer diagnostics.

## 3. Fabrication and Implementation

### 3.1. Fabrication of the Separator

**Figure 5:**
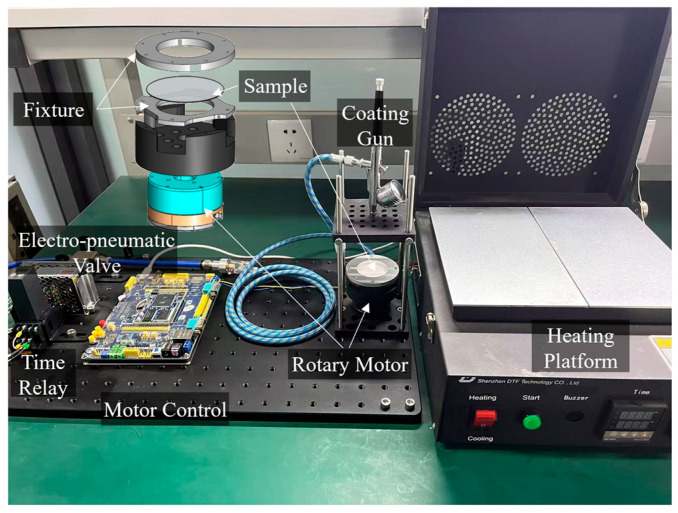
The custom-built spray-coating platform.

Following the three-layer design above, the oil–gas separator was fabricated using a custom process. The ceramic support/transition bilayer was prepared by conventional ceramic sintering and then mechanically machined into a 40 mm diameter disk. The AF2400 separation layer was subsequently deposited onto the transition layer using a custom-built spray-coating platform, as shown in Figure 5. This platform includes a rotary motor, a sample fixture, a coating gun, and a heating unit. During fabrication, the ceramic substrate was secured in the fixture, and the coating gun mounted above it enabled precise control of the spray pressure and flow rate, ensuring a uniform, consistent membrane film.

The preparation process for the separation layer is as follows. Firstly, the coating gun, ceramic substrate, and rotary stage were aligned coaxially, and the air pressure was set to 0.1 MPa. Then, 1 mL of AF2400 solution was sprayed while the substrate rotated at 100 rpm for 20 s, followed by 500 rpm for 1 min to tune the film thickness and density. After coating, the sample was left at room temperature for 10 min and then thermally treated at 165 °C for 10 min, 245 °C for 20 min, and 330 °C for 10 min, followed by natural cooling. These steps improve the film quality and enhance adhesion between the AF2400 layer and the ceramic support. It is important not to exceed 360 °C during heating, as higher temperatures can irreversibly damage the AF2400 membrane and reduce its performance.

**Figure 6:**
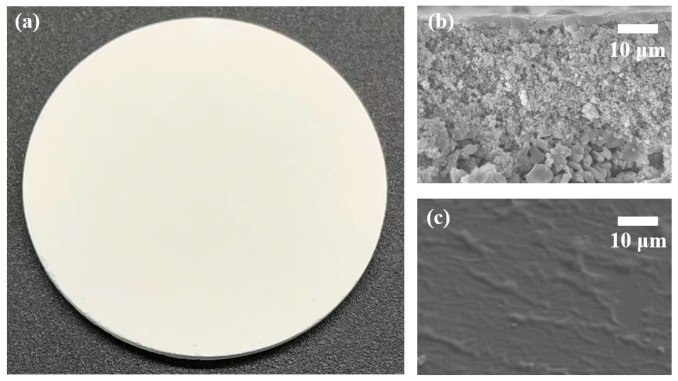
Manufactured oil–gas separator. (**a**) Photograph of the separator. (**b**) Cross-sectional SEM image of the separator. (**c**) Surface morphology of the AF2400 layer.

Figure 6 shows the fabricated membrane. The module is disk-shaped with smooth edges, as illustrated in Figure 6a. Cross-sectional SEM in Figure 6b confirms a well-bonded three-layer architecture, i.e., the separation layer (3 μm), the transition layer, and the support layer, without cracks or delamination. The AF2400 surface in Figure 6c is smooth and free of visible pores or cracks, which helps block oil-phase impurities while maintaining high gas permeability.

### 3.2. Implementation of the PAS System

**Figure 7:**
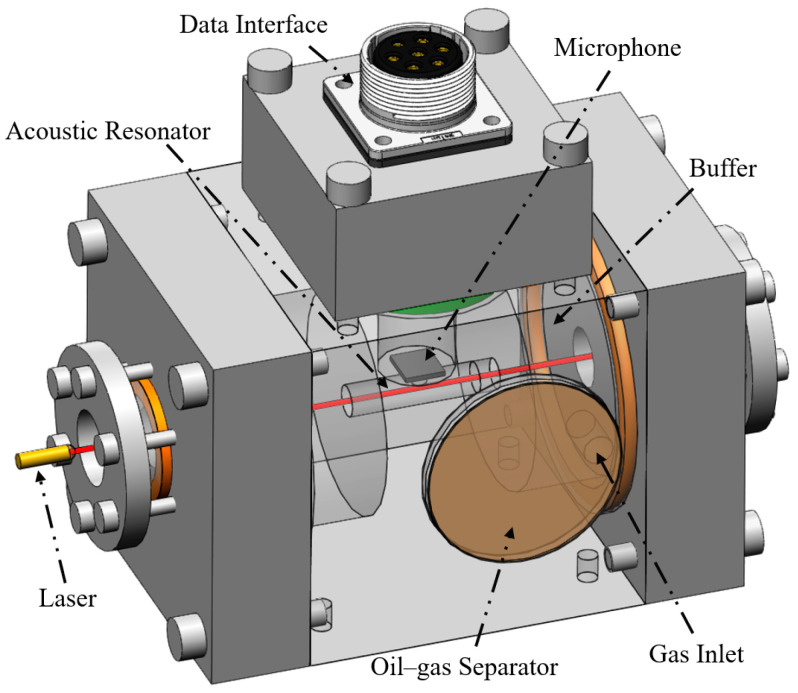
Structural design of the PAS system.

Figure 7 illustrates the structural design of the PAS system, which consists of a PAS cell, oil–gas separator, microphone, data interface, optical windows, and laser collimator. The oil–gas separation membrane is positioned in direct contact with the oil sample, allowing gas molecules from the sample to freely diffuse into the PAS cell. This membrane is securely attached to the cell using adhesive, ensuring that only purified gas samples enter the PAS cell. The gas inlet is connected to the buffer chamber, which helps dampen any fluctuations in gas flow, thereby improving the stability and signal-to-noise ratio of measurements.

Two quartz optical windows are installed on both sides of the PAS cell using flanges. The laser beam is first collimated by the laser collimator, then enters the cell through one of these windows and exits through the other. When the C_2_H_2_ gas molecules in the cell absorb the laser light, the photoacoustic signal will be generated. The PAS cell’s cavity is designed as a resonant tube, consistent with the simulation structure, which enhances the intensity of the acoustic signal. A highly sensitive microphone is positioned at the center of the resonant tube to continuously collect these subtle photoacoustic signals in real time. The acquired signals are then transmitted through the data interface to the data processing circuit for further analysis.

**Figure 8:**
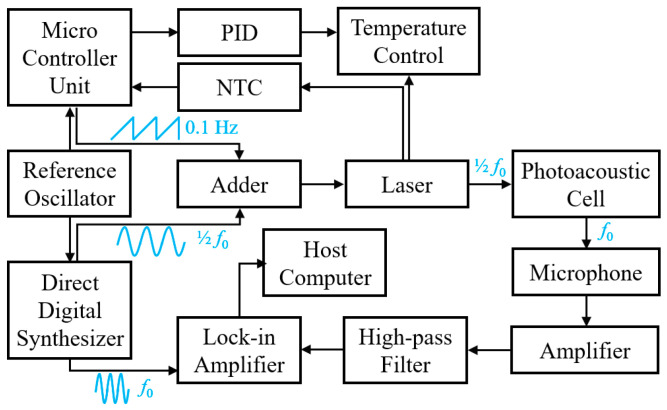
Circuit design of the system.

The electrical design of the PAS system is illustrated in Figure 8. The core of the electronic circuitry is a microcontroller unit (STM32), which is complemented by modules for laser excitation, temperature control, signal acquisition, and lock-in amplifier. The driving voltage for the laser comprises a 0.1 Hz sawtooth wave and a sine wave with a frequency of 1/2*f*_0_. The sawtooth wave is generated by the DAC module of the microcontroller unit, while the sine wave is produced by a direct digital synthesis (DDS) circuit. Both signals are combined through a low-pass filter and an adder, providing an excitation voltage for laser wavelength modulation.

The laser driver circuit is designed as a constant current source, using an operational amplifier and MOSFET to deliver stable current to the laser diode, ensuring its reliable performance. The laser integrates a negative temperature coefficient (NTC) thermistor and a thermoelectric cooler. During modulation, the temperature signal is sensed by a Wheatstone bridge and an instrumentation amplifier, then digitized via an ADC and relayed to the microcontroller. The microcontroller executes a PID temperature control algorithm, which adjusts the TEC driver circuit in response to feedback, maintaining the laser at its optimal operating temperature.

When the wavelength-modulated laser beam (modulation frequency 1/2*f*_0_) enters the photoacoustic cell, a resonant-enhanced photoacoustic signal at frequency of *f*_0_ is generated. This signal is picked up by a microphone inside the cell, and then transmitted to an amplifier and a high-pass filter to remove low-frequency noise. Subsequently, the processed signal is fed into a lock-in amplifier, which simultaneously receives a reference signal at frequency *f*_0_ generated by the DDS circuit. The lock-in amplifier extracts the weak photoacoustic signal from background noise by detecting the component at the reference frequency, which further enhances the signal-to-noise ratio of the PAS system.

## 4. Results

### 4.1. Experimental Setup

**Figure 9:**
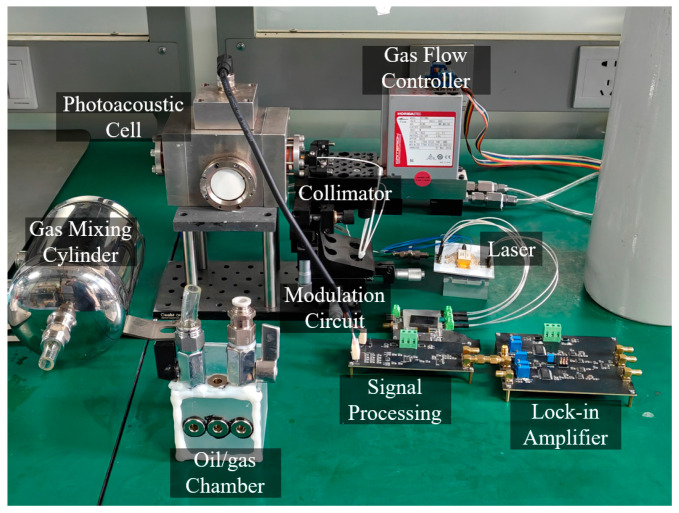
Setup of the calibration experiment.

Figure 9 presents the experimental platform developed for calibration of the carrier-free PAS system. The entire system consists of several main components: a photoacoustic cell, laser, collimator, laser modulation circuit, signal processing circuit, lock-in amplifier, and a gas distribution system. The gas distribution system utilizes two gas flow controllers (D519, HORIBA, Kyoto, Japan), a gas mixing cylinder, and an oil/gas chamber. The two gas flow controllers are connected separately to a nitrogen cylinder and an acetylene cylinder. By adjusting the flow rates of the two gases, different concentrations of acetylene can be supplied to the PAS system, thereby creating controlled acetylene atmospheres for testing.

**Figure 10:**
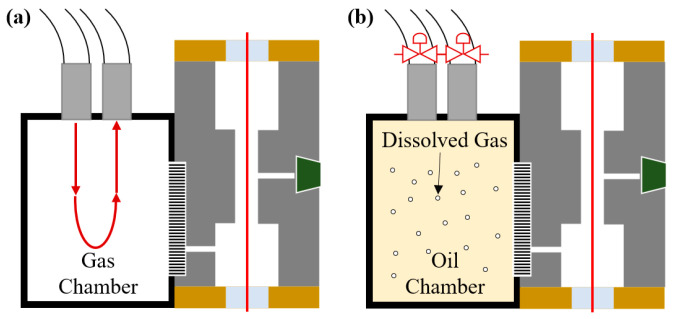
Calibration method for (**a**) gas-phase experiment and (**b**) liquid-phase experiments.

During the experiments, the oil/gas chamber is connected to the oil–gas separation module using adhesive sealing, as shown in Figure 10. This ensures that the internal gas or oil environment is in direct contact with the separation module, providing reliable and consistent sample introduction for the photoacoustic measurement.

In the gas-phase experiments, the gas concentration within the gas chamber is precisely controlled using the gas distribution system, enabling accurate sensitivity calibration of the PAS system. For the liquid-phase experiments, the oil chamber is filled with insulating oil containing 1000 ppm of dissolved acetylene. This setup is used to evaluate the system’s capability to detect acetylene dissolved in oil.

### 4.2. Eigenfrequency of the PAS Cell

In this section, the characteristic frequency of the photoacoustic cell was measured. During the experiment, the optical windows on both sides of the cell were removed, and a standard sound source was positioned directly facing the resonance tube. Driven by a signal generator, the sound source produced sinusoidal sound waves with a swept frequency, which were introduced into the photoacoustic cell. Throughout the frequency sweep, a microphone installed at the center of the resonant tube continuously monitored and recorded the resulting acoustic signals, and the peak-to-peak amplitudes of the sound waves were determined.

**Figure 11:**
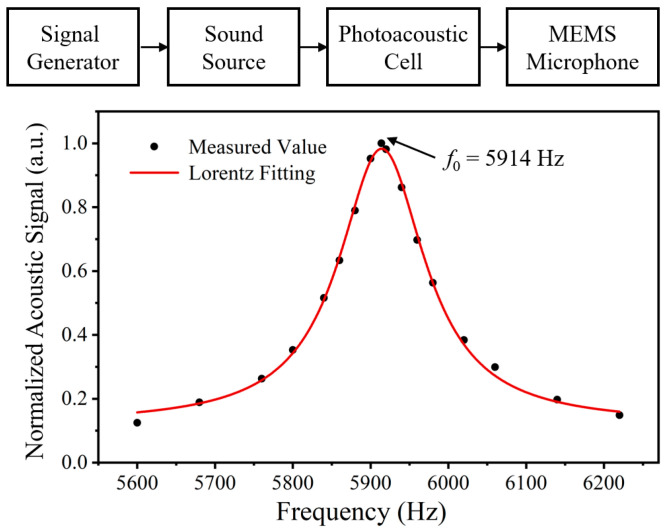
Eigenfrequency of the PAS cell.

The test results are shown in Figure 11. Within the frequency range of 5600–6220 Hz, the peak-to-peak amplitude of the sound wave initially increased and then decreased, reaching its maximum at 5914 Hz. This demonstrates that the first longitudinal mode characteristic frequency of the resonator is 5914 Hz. According to Lorentzian fitting, the FWHM of the resonance peak is 135.5 Hz, corresponding to a quality factor of 43.85. This result is in good agreement with the simulation. Therefore, the modulation frequency of the laser was set to 5914 Hz in subsequent experiments.

### 4.3. Sensitivity of the PAS System

**Figure 12:**
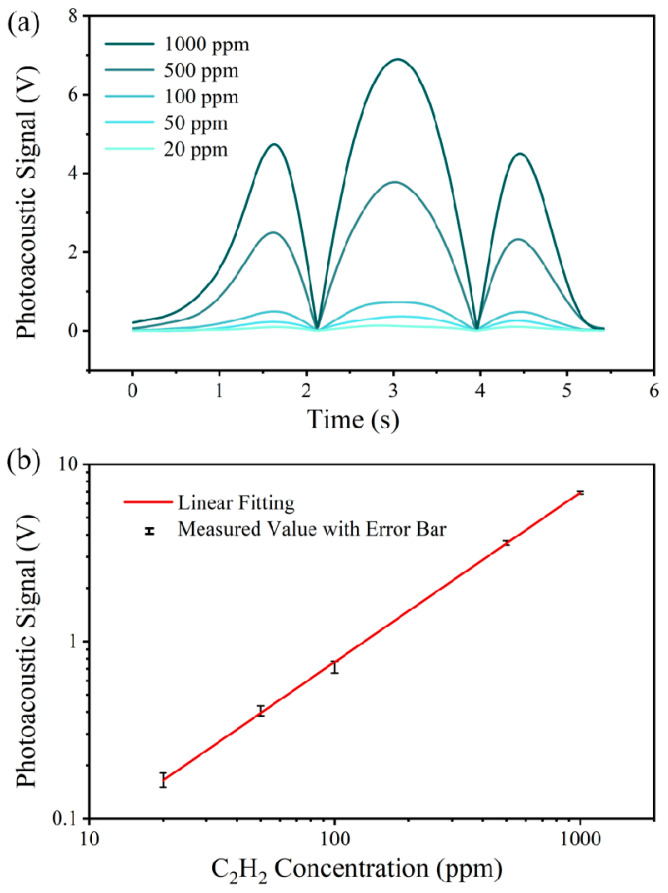
Sensitivity of the PAS system. (**a**) 2*f*-WMS signals at different concentrations. (**b**) Measured photoacoustic signal versus C_2_H_2_ concentration.

Next, the gas chamber was installed on the photoacoustic cell to perform sensitivity calibration of the PAS system. During the experiment, the gas concentration in the chamber was varied, while the corresponding output signals from the lock-in amplifier were recorded. As illustrated in Figure 12a, the maximum photoacoustic signal amplitude exhibited a clear increasing trend as the acetylene concentration increased from 20 to 1000 ppm. The sensitivity calibration experiment was repeated five times, and for each trial, the maximum amplitude of the photoacoustic signal was recorded. The collected results are presented in Figure 12b. Over the measurement range of 0–1000 ppm acetylene, the PAS system demonstrated good linearity. Linear fitting yielded a sensitivity of 6.90 mV/ppm. Based on the error bars in the figure, the system demonstrated good repeatability, while the largest deviation observed at 500 ppm was 1.65%. This indicates that the detection system maintains high accuracy and reliability across the tested concentration range.

**Figure 13:**
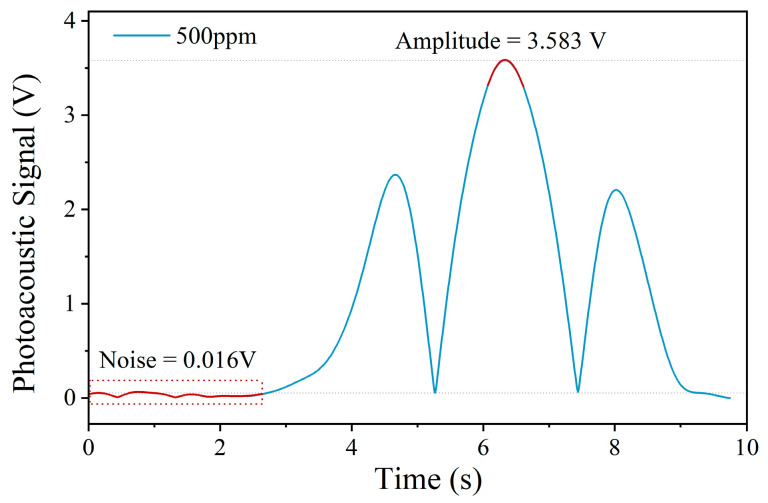
SNR of the PAS system.

Following the sensitivity calibration and linearity analysis, the minimum detection limit of the PAS system was further evaluated based on signal-to-noise analysis. During the experiment, the photoacoustic signal was measured under a fixed acetylene concentration of 500 ppm at 30 °C, as shown in Figure 13. The peak-to-peak amplitude of the photoacoustic signal was 3.583 V, while the while the noise level was approximately 0.016 V, yielding a signal-to-noise ratio (SNR) of 223.93. Based on this result, the minimum detectable concentration was estimated to be

(5) $$C_{\min}=\frac{500ppm}{SNR}=2.23ppm$$

The normalized noise equivalent absorption (*NNEA*) is another important metric for evaluating the intrinsic detection performance of a photoacoustic spectroscopy system. It can be calculated as

(6) $$NNEA=\frac{P\times \alpha }{SNR\times \sqrt{\Delta f}}$$

where *P* is the output power of the laser, *α* is the absorption coefficient, and Δf is the equivalent noise bandwidth of the lock-in amplifier. In the experiment, the laser output power was 5.4 mW, and the wavelength was tuned to the acetylene absorption line at 1521 nm. Under these conditions, the absorption coefficient was estimated to be approximately 1.475 × 10^−3^ cm^−1^, and the equivalent noise bandwidth of the lock-in amplifier was 4.7 Hz. Based on the measured signal-to-noise ratio, the *NNEA* of the proposed PAS system was calculated to be 1.640 × 10^−8^ W·cm^−1^·Hz^−1/2^.

**Figure 14:**
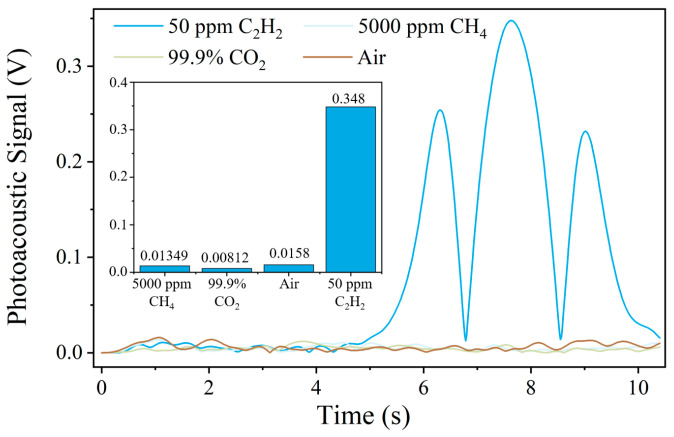
The gas selectivity of the PAS system.

In addition, the gas selectivity of the PAS system was investigated to evaluate potential cross-interference from common background gases. For this purpose, 5000 ppm CH_4_, 99.9% CO_2_, air, and 50 ppm C_2_H_2_ were individually introduced into the photoacoustic spectroscopy system under identical experimental conditions. The corresponding output signals are presented in Figure 14. It can be observed that when CH_4_, CO_2_, or air was introduced into the PAS cell, the system output remained at the noise level. In contrast, a clear signal was obtained for acetylene. These results demonstrate that the proposed PAS system exhibits good selectivity and negligible cross-interference from CO_2_, CH_4_, and air, confirming its suitability for selective acetylene detection in complex gas environments.

### 4.4. Response Time of the System

**Figure 15:**
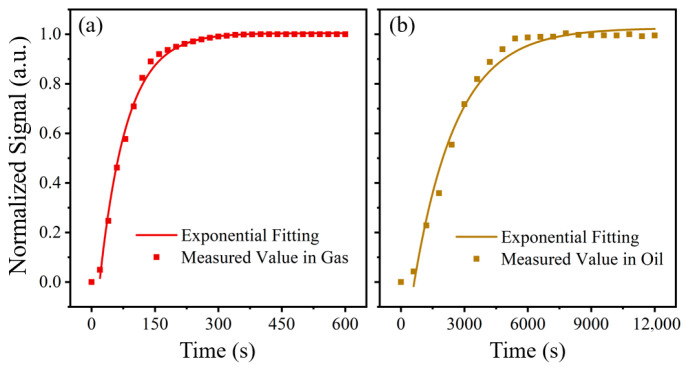
Response time of the PAS system in (**a**) gas and (**b**) insulating oil.

In the work reported in this section, the response time of the system was calibrated in both gas and liquid phases, as shown in Figure 15. In the gas-phase experiment, acetylene with a concentration of 1000 ppm was continuously introduced into the gas chamber at a flow rate of 3000 sccm. The gas entered the photoacoustic cell via free diffusion, while the PAS system recorded gas concentration data at 20 s intervals. As shown in Figure 15a, the equilibrium time *T*_90_ in the gas phase was approximately 150 s. This relatively short response is attributed to the fact that only gas molecules participate in the process, and these molecules can easily pass through the three-layer membrane structure of the oil–gas separator.

For the liquid-phase experiment, insulating oil containing 1000 ppm acetylene was directly poured into the oil chamber, fully immersing the oil–gas separator. Due to the gas permeation pressure difference on both sides of the separator, gas molecules slowly precipitated from the oil and diffused into the photoacoustic cell. In this case, the PAS system measured the gas concentration at 600 s intervals, as shown in Figure 15b. The equilibrium time was significantly prolonged to about 4350 s (72.5 min), primarily due to the slower rate of gas release from the oil. Although this equilibrium time does not outperform the conventional headspace degassing method, the PAS system offers the advantage of real-time monitoring throughout the degassing process, whereas traditional methods only allow a single measurement after degassing is complete. Therefore, this method still holds significant promise for early and rapid fault detection in transformer diagnostics.

Table 1 summarizes the proposed carrier-free PAS system with representative reported solutions, demonstrating its competitive sensitivity together with practical response characteristics for dissolved-gas sensing. Most notably, it achieves a response time of 72.5 min under a passive diffusion condition, without the use of an oil pump. This response time is substantially shorter than that of commercial FEP-based separators [23,32], and even surpasses the active circulation approach reported in [33], which requires 120 min. These results confirm the practical significance and superior efficiency of the proposed design for in situ DGA applications.

## 5. Conclusions

In this study, a carrier-free PAS system was developed for the real-time monitoring of dissolved acetylene in oil. An optimized oil–gas separation membrane was designed and fabricated using a custom-built spray-coating platform. On a testing circuit, a lock-in amplifier for precise signal acquisition was implemented for the PAS system. According to experimental results, the sensitivity of the system is 6.90 mV/ppm, with a maximum repeatability error of 1.65%. Response time tests indicated that the PAS system achieved rapid equilibrium in the gas phase (*T*_90_ = 150 s) and the liquid phase (*T*_90_ = 72.5 min). Compared to traditional headspace degassing methods, the PAS approach enables continuous, real-time measurements throughout the degassing process, thus supporting quicker fault detection.

## Figures and Tables

**Table 1:** Performance comparison between this work and representative reported solutions.

Reference	Thickness	Separator Material	Diffusion Mode	Response Time
[20]	N/A	Teflon AF2400 membrane	Without oil pump	720 min
[23]	12.5 μm	Commercially available FEP film	Without oil pump	144 min
[32]	30 μm	Commercially available FEP film	Without oil pump	378 min
[33]	1.1 μm	Teflon/PAI composite membrane	With oil pump	120 min
This work	3 μm	Custom-fabricated AF2400-coated ceramic membrane	Without oil pump	72.5 min

## Data Availability

The data presented in this study are available on request from the corresponding author.

## Abbreviations

The following abbreviations are used in this manuscript:

DGA	Dissolved gas analysis
PAS	Photoacoustic spectroscopy
DDS	Direct digital synthesis
NTC	Negative temperature coefficient
FWHM	Full width at half maxima
